# Editorial Comment from Dr Sadahira and Dr Tsuboi to Testicular sarcoidosis with bilateral scrotal swelling

**DOI:** 10.1002/iju5.12127

**Published:** 2019-10-30

**Authors:** Takuya Sadahira, Ichiro Tsuboi

**Affiliations:** ^1^ Department of Urology Dentistry and Pharmaceutical Sciences Okayama University Graduate School of Medicine Okayama Japan

Genitourinary sarcoidosis is extremely rare, and only a few cases of bilateral testicular sarcoidosis have been reported in Japan. The differential diagnosis of scrotal masses includes testicular cancer. Serum parameters can be helpful in distinguishing a scrotal mass from testicular cancer, but a testicular biopsy is currently required to completely rule out malignancy. Kimura *et al*.^1^ reported a rare case of bilateral testicular sarcoidosis, which can also be diagnosed based on biopsy findings. Although a radical orchiectomy was selected as treatment for testicular sarcoidosis in previous case reports because of the possible association between sarcoidosis and testicular malignancy, orchiectomy should be considered for patients in whom no alternatives are available or in whom a malignancy is suspected, especially in young men.^2^ Recently, Konishi *et al*.^3^ concluded that the lectin array assay is a less invasive tool for distinguishing sarcoidosis from a malignancy, thus the lectin array assay has a potential to find biomarkers for sarcoidosis. Further research is warranted to verify this finding.

The periductal distribution of granulomas might cause ductal compression or Leydig cell damage.^4^ In some cases, young patients with testicular sarcoidosis have oligospermia or azoospermia. Systematic corticosteroid therapy may improve the sperm count following regression of a space‐occupying granuloma; however, the dose and length of this therapy are controversial. Further studies are necessary to confirm these findings.

## Conflict of interest

The authors declare no conflict of interest.
